# Treatment effects of renin-angiotensin aldosterone system blockade on kidney failure and mortality in chronic kidney disease patients

**DOI:** 10.1186/s12882-017-0753-9

**Published:** 2017-11-29

**Authors:** Phisitt Vejakama, Atiporn Ingsathit, Gareth J. McKay, Alexander P. Maxwell, Mark McEvoy, John Attia, Ammarin Thakkinstian

**Affiliations:** 1 Section for Clinical Epidemiology and Biostatistics, Faculty of Medicine, Ramathibodi Hospital, Mahidol University, Bangkok, Thailand; 2 Department of Medicine, Sunpasitthiprasong Hospital, Province, Ubon Ratchathani, Thailand; 3 Centre for Public Health, Queen’s University Belfast, Belfast, Northern Ireland; 4 Centre for Clinical Epidemiology and Biostatistics, School of Medicine and Public Health, Faculty of Health and Medicine, University of New Castle, NSW, Australia

## Abstract

**Background:**

Chronic kidney disease (CKD) is a leading cause of death before and after onset of end-stage renal disease (ESRD). Knowing treatments that can delay disease progression will lead to reduced mortality. We therefore aimed to estimate the effectiveness of renin angiotensin aldosterone system (RAAS) blockade on CKD progression.

**Methods:**

We conducted a retrospective CKD cohort at Ubon Ratchathani province, Thailand from 1997 to 2011. ESRD was defined as estimated glomerular filtration rate (eGFR) <15 ml/min/1.73 m2, dialysis, or kidney transplantation. All-cause mortality was verified until December 31, 2011. A counterfactual-framework was applied to estimate the effectiveness of RAAS blockade on outcomes, i.e., ESRD, death before and after ESRD. RAAS blockade was categorized according to duration of use <0.25 year, 0.25–1 year (RAAS1), and >1 year (RAAS2). An augmented inverse-probability weighting (AIPW) method was used to estimate potential-outcome mean (POM) and average treatment-effect (ATE). Multi-logit and Poisson regressions were used for treatment and outcome models, respectively. Analyses were stratified by ESRD, death before/after ESRD for diabetic and non-diabetic groups. STATA 14.0 was used for statistical analyses.

**Results:**

Among 15,032 diabetic patients, 2346 (15.6%), 2351 (18.5%), and 1607 (68.5%) developed ESRD, died before ESRD, and died after ESRD, respectively. Only RAAS2 effect was significant on ESRD, death before and after ESRD. The ESRD rates were 12.9%, versus 20.0% for RAAS2 and non-RAAS, respectively, resulted in significant risk differences (RD) of −7.2% (95% CI: -8.8%, −5.5%), and a numbers needed-to-treat (NNT) of 14. Death rates before ESRD for these corresponding groups were 14.4% (12.9%, 15.9%) and 19.6% (18.7%, 20.4%) with a NNT of 19. Death rates after ESRD in RAAS2 was lower than non-RASS group (i.e., 62.8% (55.5%, 68.9%) versus 68.1% (65.9%, 70.4%)) but this was not significant. RAAS2 effects on ESRD and death before ESRD were persistently significant in non-diabetic patients (*n* = 17,074) but not for death after ESRD with the NNT of about 15 and 16 respectively.

**Conclusions:**

Receiving RAAS blockade for 1 year or longer could prevent both CKD progression to ESRD and premature mortality.

**Electronic supplementary material:**

The online version of this article (10.1186/s12882-017-0753-9) contains supplementary material, which is available to authorized users.

## Background

Chronic kidney disease (CKD) is one of the leading non-communicable diseases contributing to morbidity and mortality globally. Hypertension and diabetes are the strong predictors for the development and progression of chronic kidney diseases [1–4]. Experimental study reveals histologic and immunohistochemical similarities between the evolving fatty streak in the atherosclerotic vessel wall and the progressive glomerular lesion leading to glomerulosclerosis suggest analogous pathobiologic mechanisms [5]. The role of RAAS in the pathogenesis of cardiorenal disease is widely accepted. The kidney contain all elements of the RAAS, and intrarenal formation of angiotensin II not only controls glomerular hemodynamics and tubule sodium transport, but also activated inflammatory and fibrotic pathways [6].

Furthermore, estimates of age-standardized mortality from the Global Burden of Disease 2013 study have ranked CKD highest in terms of years of life lost for any non-communicable cause of premature death between the years 1990 and 2013 [7]. Current guidelines identify individuals with CKD as being at high risk for cardiovascular disease (CVD) and other adverse outcomes and that individuals who succumb to end-stage renal disease (ESRD) have a very high mortality, often as a consequence of accelerated CVD [8]. The primary therapeutic options to prevent and treat CKD are blood pressure control, mainly through renin angiotensin aldosterone system (RAAS) blockade, i.e. angiotensin converting enzyme inhibitors (ACEi), angiotensin receptor blockers (ARB), combined with improved glycemic control.

Previous evidences from meta-analyses of randomized controlled trials (RCTs) have been approved efficacy of RAAS blockade in prevention of CKD occurrence in type 2 diabetes [9], delay progression of heart failure [10], all-cause-mortality [11], and ESRD in CKD patients [12]. Nevertheless, a number of observational studies have reported contrasting findings for the effectiveness of RAAS blockade in terms of CKD progression to ESRD and mortality [13–15]. In addition, many of the observational studies assessing efficacy in reducing premature mortality were performed in clearly defined subject groups (e.g. patients with CVD [16–22], diabetes [23], and hypertension [14]), with only a single study [24] evaluating subjects with variable phenotypic categories based on GFR/albuminuria. As such, the application of these findings to the general population may be questionable. Traditionally, observational studies are considered to be subject to confounding and until well designed, RCTs are performed for this specific question in this particular population, the evidence for treatment effectiveness is considered to be uncertain.

However, new methodologic developments in recent years have increased the arsenal of statistical tools for limiting the effects of confounding in observational studies. One such tool is counterfactual analysis, which allows the evaluation of treatment effectiveness in clinical practice or in an area where an RCT cannot be conducted (i.e., treatment intervention cannot be randomly allocated) for ethical reasons or due to high cost, which has been used in health sciences [25–27] and economic research [28]. An outcome model using counterfactual analysis is constructed to estimate treatment effects at the individual-patient level, when patients do and do not receive treatments. The treatment effect is the change of outcome value when an individual patient receives one or another treatment. This treatment effect at the individual-patient level cannot be directly observed because individual patients receive only one treatment once at a time. For instance, the outcome of patients who do not receive treatment are actually observed as Y0s, while their potential outcomes would be Y1s if they received treatment. Thus, Y1s are the potential outcome or counterfactuals for these patients. For patients who do receive treatment, their actual Y1s are then observed, and their counterfactual outcomes, Y0s, are estimated.

We apply this method to answer our research questions: Does RAAS blockade delay CKD progression to ESRD and death in diabetic and non-diabetic patients? Does RAAS blockade still work after ESRD development? We therefore conducted a retrospective cohort study to assess the effectiveness of RAAS blockers in prevention of ESRD, and premature death before and after ESRD in both diabetic and non-diabetic CKD patients using counterfactual analysis.

## Methods

A retrospective cohort of CKD subjects was retrieved from 20 districts of Ubon Ratchathani province, in North Eastern Thailand; details have been published previously [29]. Briefly, population screening tests for hypertension, diabetes, dyslipidemia, CVD, along with laboratory tests (i.e., CBC, fasting plasma glucose (FPG), lipid profile, blood urea nitrogen (BUN) assay, serum creatinine, and urine analysis (UA)) were performed annually. Data for these screening programs from 1997 to 2011 were retrieved electronically. Serum creatinine was used to estimate glomerular infiltration rate (eGFR) using the CKD-EPI equation [30]. CKD was then classified according to the Kidney Disease: Improving Global Outcome (KDIGO) 2012 guideline [31]. All consecutive CKDs were then retrieved from the whole database and kept for analysis. In addition, individual diagnosis of CKD was verified by merging this survey data with district hospital database for each catchment area in the studied province. CKD subjects were considered eligible for this study if they were 18 years or older, and had at least 3 months of follow-up.

### Variables and measurements

eGFR was categorized into 6 main categories according to the KDIGO 2012 guideline [31], which were normal or high (≥90 ml/min/1.73 m^2^, G1), mildly decreased (60–89 ml/min/1.73 m^2^, G2), mildly to moderately decreased (45–59 ml/min/1.73 m^2^, G3a), moderately to severely decreased (30–44 ml/min/1.73 m^2^, G3b), severely decreased (15–29 ml/min/1.73 m^2^, G4), and ESRD (<15 ml/min/1.73 m^2^, G5). We used only outpatient determination of serum creatinine to avoid acute changes due to inter-current illness and we ascertained the chronicity of ESRD as an eGFR <15 ml/min/1.73 m^2^ for 3 months or more. eGFR was repeatedly assessed at least once or twice yearly during follow up depending on patients’ condition.

Albuminuria was assessed using a urine dipstick for testing urine protein (CYBOW ™, Gyung-Num, Republic of Korea). Albuminuria was categorized as normal to mild (urine protein reagent strip negative to trace, equivalent to albumin-to-creatine ratio (ACR) < 30 mg/g, A1), moderate (urine protein reagent strip +, ACR 30–300 mg/g, A2), and severe (urine protein reagent strip ≥ ++, ACR > 300 mg/g, A3).

Data for RAAS blockade (i.e., ACEI and ARB) including prescriptions (i.e., dosage, amount, and prescribed date) were retrieved from the hospital databases and merged with survey databases. These follow-up data allowed us to calculate duration of RAAS use for individual subjects during the study period. This variable was then categorized as non-RAAS user for those subjects who had never used or used for less than 3 months, RAAS group 1 (called RAAS1) for those subjects who ever used for 3 months to 1 year, and RAAS group 2 (called RAAS2) for those who had used longer than 1 year.

Diabetes, hypertension, and CVD at diagnosis of CKD were identified from the databases according to the ICD10. Diagnosis of diabetes was defined as fasting plasma glucose ≥126 mg% on at least 2 separate occasions. This diagnosis was also validated by prescriptions of anti-diabetic medications. Hypertension was defined by systolic blood pressure at rest ≥140 mmHg or diastolic blood pressure ≥ 90 mmHg. CVD included any of following diseases: coronary artery disease (i.e., ST elevation myocardial infarction, non-ST elevation myocardial infarction, unstable angina, and chronic stable angina), and cerebrovascular disease (i.e., cerebral infarction or hemorrhage, and transient ischemic attack).

### Primary outcomes

The primary outcomes of interest were ESRD and death prior to or after onset of ESRD. ESRD was defined according to the KDIGO 2012 guideline [31] as eGFR <15 ml/min/1.73m^2^ persisting for 3 months or longer, or being on chronic dialysis treatment for 3 months or longer, or having had a kidney transplant. All-cause mortality was verified from the death certificate database at the Bureau of Strategy and Statistics, Ministry of Public Health, through to December 31, 2011. Since we were able to capture all deaths in our cohort, we assumed that there was no loss to follow up.

### Statistical analysis

A total of 32,106 CKD subjects were included in the main analyses, which consisted of 15,032 (46.8%) and 17,074 (53.2%) diabetic and non-diabetic subjects. Data for body weight, height, and albuminuria at baseline were missing in 0.5%, 12.4%, and 25%, respectively. Missing data were imputed using multivariate chain equations assuming that data were missing at random [32, 33]. Twenty imputations were constructed using linear regression to obtain the summarized estimates for further analysis [34]. Characteristics of CKD patients were described using mean (SD) and frequency (percentage) for continuous and categorical data, respectively.

Treatment effectiveness was determined separately by diabetic and non-diabetic groups using a counterfactual framework approach as follows [35, 36]. First, probability of receiving treatment was estimated using a multi-logit treatment model (also known as treatment assignment process) by fitting treatment variable (RAAS1 and RAAs2 vs non-RAAS groups) on co-variables including age, gender, body mass index (BMI), diabetes, hypertension, cardiovascular disease, baseline eGFR, baseline albuminuria, and high density lipoprotein (HDL). Second, the outcome model was constructed using Poisson regression with augmented inverse-probability weights (AIPW). Potential outcome values (i.e., Y0 and Y1) for individual patients were then estimated according to treatment assignment process. These values were averaged, called potential outcome means (POM), by RAAS groups. Finally, average treatment effect (ATE), differences in POMs by RAAS groups were estimated.

These analyses were stratified by ESRD, and death before or after ESRD onset. The same covariables were adjusted in the outcome model, except eGFR was not included in ESRD model because it was the same value used for diagnosing ESRD. STATA version 13.1 was used for statistical analyses [37]. A *p*-value threshold of 0.05 was used for statistical significance.

## Results

A total of 32,106 out of 195,706 patients were classified as having CKD according to criteria of GFR and albuminuria. Among them, 15,032 (46.8%) and 17,074 (53.2%) patients were diabetic and non-diabetic patients, and their characteristics of patients were described, see Table 1. Among diabetic group, mean age and BMI were 61 (SD = 11.0) years and 23.7 (SD = 4.0) kg/m^2^ respectively, and the majority were females (73.0%). The percentages of hypertension and CVD were 47.0%, and 11.0%, respectively. Their mean GFR was 48 (SD = 19) ml/min/1.73 m^2^ with median follow up time of 4.7 years. About 30% of patients received RAAS blockade, in which majority (99.5%) received ACEi with a median of use of 3.1 years. For those received RAAS blockade, about 4% received RAAS blockade shorter than 3 months (RAAS1) and 26% received 3 months or longer (RAAS2). Among RAAS1 group, 30.0%, 26.9%, and 13.0% used diuretics, calcium channel blockers, and beta blockers, respectively. Among RAAS2 group, 33.7%, 30.7%, and 14.4% used diuretics, calcium channel blockers, and beta blockers, respectively. Patients in non-diabetic groups were a little older but lower BMI and female percentage than diabetic groups with means age, BMI, and percent females of 65.7 (SD = 13.9), 21.8 kg/m2, and 55%, respectively. Comorbidities were not much different in non-diabetes when compared diabetic groups with percent hypertension and CVDs of 39% and 16%. Percent receiving RAAS blockade in non-diabetic group was nearly a half (i.e., 14.6%) lower than diabetic group with a median use of 2.7 years. Their mean GFR was 46 (SD = 22) ml/min/1.73 m^2^ with a median follow up time of 4.2 years. The patients’ baseline characteristics between RAAS groups for both diabetic and non-diabetic groups were also described in Table 1. All patient characteristics were statistically significant between RAAS1, RAAS2, and non-RAAS groups.

**Table 1:** Describe baseline characteristics of subjects

			Diabetic group					Non-diabetic group		
Variable	Total	Non-RAAS	RAAS1	RAAS2	*P*-value	total	Non-RAAS	RAAS1	RAAS2	*P*-value
Sample Size	15,032	10,560	623	3849		17,074	14,587	588	1899	
Age	61.0 (11.0)	61.0 (11.2)	63.2 (10.9)	60.6 (10.6)	<0.001	65.7 (13.9)	65.5 (14.3)	68.2 (11.4)	66.4 (10.8)	<0.001
Male	4048 (27)	2671 (25)	220 (35)	1157 (30)	<0.001	7659 (45)	6541 (45)	269 (46)	849 (45)	0.901
BMI	23.7 (4.0)	23.4 (4.0)	23.4 (3.9)	24.5 (4.0)	<0.001	21.8 (4.3)	21.5 (4.3)	22.5 (4.1)	23.2 (4.4)	<0.001
SBP	128.0 (20.2)	124.0 (19.8)	127.7 (20.6)	131.1 (19.7)	<0.001	127.8 (23.9)	124.4 (23.1)	135.1 (23.8)	137.3 (23.5)	<0.001
DBP	77.4 (11.4)	75.7 (11.3)	77.3 (11.3)	78.8 (11.4)	<0.001	77.4 (17.4)	75.9 (18.5)	80.3 (13.6)	81.6 (13.4)	<0.001
Comorbidity										
Hypertension	7059 (47)	3632 (34)	480 (77)	2947 (77)	<0.001	6742 (39)	4466 (31)	510 (87)	1766 (93)	<0.001
CVD	1712 (11)	1129 (11)	98 (16)	485 (13)	<0.001	2659 (16)	2108 (14)	122 (21)	429 (23)	<0.001
HDL	37.7 (11.0)	37.4 (10.8)	37.3 (10.5)	38.5 (11.5)	<0.001	40.2 (9.0)	40.0 (8.6)	40.8 (10.7)	41.6 (11.2)	<0.001
LDL	115.3 (40.5)	114.6 (39.9)	114.8 (40.0)	117.3 (42.1)	0.002	115.2 (32.8)	114.2 (31.2)	119.7 (39.3)	121.0 (41.2)	<0.001
Albumin										
A1	8657 (58)	5975 (57)	383 (61)	2299 (60)	<0.001	11,079 (65)	9133 (63)	432 (73)	1514 (80)	<0.001
A2	3367 (22)	2499 (24)	100 (16)	768 (20)	<0.001	3485 (20)	3188 (22)	77 (13)	220 (12)	<0.001
A3	3008 (20)	2086 (20)	140 (22)	782 (20)	<0.001	2510 (15)	2266 (16)	79 (13)	165 (9)	<0.001
GFR	48.0 (19.0)	47.1 (19.4)	42.7 (18.3)	51.2 (17.6)	<0.001	46.0 (22.5)	45.9 (23.5)	41.4 (16.5)	47.9 (14.2)	<0.001
FU time^*^	4.7(0.3–13.2)	4.9 (0.3–14.2)	2.6 (0.3–11.9)	4.5 (1.0–13.9)	<0.001	4.2(0.3,14.3)	4.3(0.2–4.3)	3.0(0.3–3.7)	4.2(1.0–13.3)	<0.001

Values are expressed as mean (SD) for continuous data, except *median (range), number (%) for dichotomous data

*RAAS* Renin-angiotensin aldosterone system, *RASS 1 & 2* Duration = 0.25–1 & > 1 year, *CVD* Cardiovascular diseases

### Effects of RAAS blockade on ESRD

The treatment and ESRD models were constructed separately for diabetic and non-diabetic groups using multi-logit and Poisson models with the AIPW method, see Additional file 1: Table S1-S2. All factors (i.e., age, gender, BMI, hypertension, CVD, HDL, albuminuria, and eGFR,) were associated with receiving RAAS blockers in non-diabetic and non-diabetic patients, except CVD that was not significantly associated in diabetic group.

Among 15,032 diabetic patients, 2346 (15.6%) patients developed ESRD, 1607 (68.5%) patients died after ESRD, whereas 2351 (18.5%) patients died without developing ESRD. These later patients were not included in the analysis of RAAS blockade on ESRD. The risks of having ESRD were 12.9%, 19.0%, and 20.0% in RAAS2, RAAS1, and non-RAAS groups, respectively. The risk difference (RD) was statistically significant for only RAAS2 but not for RAAS1 with the RDs of −7.1% (95% CI: -8.8%,-5.5%) and −1.0% (95% CI: -4.5%, 2.5%) for RAAS2 and RAAS1 versus non-RAAS groups, respectively (see Table 2). As a results, the estimated number needed to treat (NNT) for RAAS2 was 14 (95% CI: 11, 17), i.e., about 14 CKD patients with diabetic would have to be treated with RAAS blockade for longer 1 year in order to prevent 1 ESRD.

**Table 2:** Estimation of average treatment effects and potential outcome mean of RAAS blockade on ESRD by diabetic groups

Model for diabetic patients	Treatments	RD	LL.	UL	NNT	LL	UL
ATE	RAAS1 vs non-RAAS	−0.0100	−0.0445	0.0246	−100.3	−447.6	247.0
	RAAS2 vs non-RAAS	−0.0712	−0.0878	−0.0547	−14.0	−17.3	−10.8
		Risk	LL	UL	RR	LL	UL
POM	Non-RAAS	0.1999	0.1917	0.2082	1		
	RAAS1	0.1899	0.0779	0.1905	0.950	0.778	1.123
	RAAS2	0.1287	0.1139	0.1435	0.644	0.567	0.721
Model for non-diabetic patients	Treatments	RD	LL.	UL	NNT	LL	UL
ATE	RAAS1 vs non-RAAS	−0.0205	0–.0863	0.0453	−48.7	−204.9	107.5
	RAAS2 vs non-RAAS	−0.0674	−0.1216	−0.0132	−14.8	−26. 8	−2.9
		Risk	LL	UL	RR	LL	UL
POM	Non-RAAS	0.1812	0.1745	0.1879	1		
	RAAS1	0.1607	0.0949	0.2265	0.887	0.523	1.250
	RAAS2	0.1139	0.0598	0.1679	0.628	0.3301	0.926

*ATE* Average treatment effect, *LL* Lower limit, *POM* Potential outcome mean, *RAAS1* Duration use of 0.25–1 year, *RAAS2* Duration use of >1 year, *RD* Risk difference, *RR* Rate ratio, *UL* Upper limit

For 17,074 non-diabetic patients, 2442 (14.2%) patients developed ESRD, 11,427 (66.9%) patients stayed with ESRD free, and 3225 (18.9%) patients died before ESRD development. Thus, these 3225 patients were not included in analysis of ESRD. Risks of having ESRD were 11.4%, 16.1%, and 18.1% for RAAS2, RAAS1, and non-RAAS groups, see Table 2. The RD was significant for only RAAS2 versus non-RAAS with the RD of −6.7% (95% CI: -12.2%, -1.3%) and the NNT of 15 (95% CI: 3, 27). This could be interpreted that about 15 CKD patients without diabetes are needed to be treated to prevent 1 ESRD.

### RAAS blockade effects on risk of death prior to onset of ESRD

A total of 2351/15,032 (15.5%) and 3225/17,074 (18.8%) patients with and without diabetes died before developing ESRD. Risk of death prior ESRD were much similar between diabetic and non-diabetic groups, i.e., 14.4%, 22.7%, and 19.6% for RAAS2, RAAS1, and non-RAAS groups for diabetic group; and 15.9%, 22.4%, and 22.3% for non-diabetic groups, see Table 3. The RDs were statistically significant for only RAAS2 versus non-RAAS, i.e., −5.2% (95% CI: -6.9%, −3.5%) and −6.4% (95% CI: -9.9%, −2.9%) for diabetic and non-diabetic patients, respectively. As a result, the NNTs for these corresponding groups were approximately 19 (95% CI: 13, 26) and 16 (95% CI: 7, 24).

**Table 3:** Estimation of average treatment effects and potential outcome mean of RAAS treatment on death before ESRD by diabetic groups

Model for diabetic patients	Factors	RD	LL	UL	NNT	LL	UL
ATE	RAAS1 vs non-RAAS	0.030	−0.012	0.072	32.9	−12.6	78.5
	RAAS2 vs non-RAAS	−0.052	−0.069	−0.035	−19.1	−25.5	−12.9
		Risk	LL	UL	RR	LL	UL
POM	Non-RAAS	0.196	0.187	0.204			
	RAAS1	0.227	0.186	0.268	1.155	0.939	1.369
	RAAS2	0.144	0.129	0.159	0.773	0.652	0.815
Model for non-diabetic patients	Factors	RD	LL	UL	NNT	LL	UL
ATE	RAAS1 vs non-RAAS	−0.009	−0.066	0.048	−113.6	−848.7	621.5
	RAAS2 vs non-RAAS	−0.064	−0.099	−0.029	−15.6	−24.2	−7.1
		Risk	LL	UL	RR	LL	UL
POM	Non-RAAS	0.223	0.215	0.229			
	RAAS1	0.224	0.157	0.270	0.960	0.705	1.216
	RAAS2	0.159	0.124	0.193	0.713	0.557	0.868

*ATE* Average treatment effect, *LL* Lower limit, *POM* Potential outcome mean, *RAAS1* Duration use of 0.25–1 year, *RAAS2* Duration use of >1 year, *RD* Risk difference, *RR* Rate ratio, *UL* Upper limit

### RAAS blockade effects on risk of death after onset of ESRD

Among 2346 and 2442 ESRD patients with diabetes and without diabetes, 1607 (68.5%) and 973 (59.8%) patients died after ESRD diagnosis. Risks of death by RAAS groups were estimated (see Table 4), indicating lower risk for RAAS2 but higher risk for RAAS1 when compared with non-RAAS in both patients groups. However, these effects were not statically significant.

**Table 4:** Estimation of average treatment effects and potential outcome mean of RAAS treatment on death after ESRD by diabetic groups

Model for diabetic patients	Treatments	RD	LL	UL	NNT	LL	UL
ATE	RAAS1 vs non-RAAS	0.062	−0.037	0.161	16.1	−9.5	41.7
	RAAS2 vs non-RAAS	−0.059	−.0129	0.0112	−16.9	−36.9	3.18
		Risk	LL	UL	RR	LL	UL
POM	Non-RAAS	0.681	0.659	0.704	1		
	RAAS1	0.743	0.647	0.839	1.091	0.945	1.236
	RAAS2	0.622	0.555	0.689	0.913	0.810	1.016
Model for non-diabetic patients	Treatments	RD	LL	UL	NNT	LL	UL
ATE	RAAS1 vs non-RAAS	0.124	−0.059	0.306	8.1	−3.8	19.9
	RAAS2 vs non-RAAS	−0.121	−0.333	0.092	−8.3	−22.9	6.3
		Risk	LL	UL	RR	LL	UL
POM	Non-RAAS	0.596	0.576	0.617	1		
	RAAS1	0.719	0.539	0.901	1.209	0.909	1.509
	RAAS2	0.475	0.264	0.687	0.798	0.435	1.162

*ATE* Average treatment effect, *LL* Lower limit, *POM* Potential outcome mean, *RAAS1* Duration use of 0.25–1 year, *RAAS2* Duration use of >1 year, *RD* Risk difference, *RR* Rate ratio, *UL* Upper limit

## Discussion

We have assessed the effectiveness of RAAS blockers using data from an observational CKD cohort. Our results suggest that use of RAAS blocking drugs for longer than 1 year in patients with CKD reduces major clinical outcomes, i.e., ESRD progression and death prior ESRD onset in both diabetic and non-diabetic patients. For every 100 diabetic and non-diabetic subjects with CKD being treated with RAAS blockers for longer than 1 year, approximately 14 and 7 cases of ESRD, 5 and 6 deaths before ESRD will be prevented over 4.5 years. We found no association between death after ESRD and RAAS blockers treatment among both diabetic and non-diabetic CKD patients.

Chronic RAAS inhibition using ACEi or ARBs as mono-therapy is the standard of care to treat the vaso-constrictive/pro-inflammatory effects of RAAS activation as recommended in the revised 2014 American Diabetes Association guidelines for patients with micro-albuminuria or macro-albuminuria [38]. The pharmacological mechanism of RAAS blockade in the progression of kidney disease is likely through the hemodynamic effects and blood pressure control [39]. Drugs that block RAAS have been shown to reduce proteinuria while managing hypertension and reducing cardiovascular risk [40]. A recent meta-analysis of mortality reduction with RAAS blockade in hypertension demonstrated a 10% reduction in all-cause mortality with a trend towards a 12% reduction in cardiovascular mortality with ACEi, but no significant reduction in mortality with ARBs [41]. RAAS inhibition in hypertensive patients with ACEi has been reported to decrease central aortic BP, reduce arterial stiffness and lessen cardiovascular events [42].

The effectiveness of RAAS blockade in preventing CKD progression to ESRD among diabetic CKD patients is consistent with findings from a recent meta-analysis of RCTs [9] evaluating their reno-protective efficacy in type 2 diabetic subjects which demonstrated approximately 23% (RR = 0.77, 95% CI: 0.64, 0.92) reduction of ESRD risk. The results of our study were within the range of these pooled data and thus support the true effects of RAAS blockade on ESRD. In contrast, a nested case-control design [13] reported increasing ESRD after 3 years among diabetics using RAAS-blockade. Although this study adjusted for some confounding variables, there was likely residual confounding, either observed or unobserved. The ‘counterfactual’ approach employed here estimates what would have happened to beneficiaries in the absence of the intervention, with the impact estimated by comparing counterfactual outcomes to those observed under the intervention. The key challenge for impact evaluation in this instance is that the counterfactual approach cannot be directly observed and must be estimated with reference to a comparison group in an observational study.

It was known that diabetic CKD subjects have different prognosis from non-diabetic CKD patients because they progress about twice as rapidly as non-diabetic subjects to reach ESRD so the beneficial effect of RAAS blockage has to consider specifically among non-diabetic subjects [43]. One possibility mechanism is that chronic hyperglycemia alters vasoactive regulators of afferent and efferent arteriolar tone, leading to increased glomerular capillary pressure, hyperperfusion and hyperfiltration [44]. The activation of the RAAS is considered to be the key factor causing glomerular vascular dysfunction, sodium and fluid retention, hypertension, increased intraglomerular pressure and inflammation, all of which contribute to end-organ damage [45, 46]. The evidence of RAAS blockage as the initial treatment of hypertension for patients with diabetic CKD is relatively robust and based on RCTs with hard renal outcomes. [47–50]. However, in patients with non-diabetic CKD, the evidence in favor of a particular class of anti-hypertensive drugs is much more limited.

A meta-analysis of patient-level data of 1860 non-diabetic CKD patients revealed antihypertensive regimens that include ACEi are more effective than regimens without ACE inhibitors in slowing the progression of non-diabetic renal disease which demonstrated relative risk in the ACEi group was 0.69 (CI, 0.51 to 0.94) for end-stage renal disease [51]. This finding was supported by more recent RCT, the African American Study of Kidney Disease and Hypertension (AASK) trial which was conducted among hypertensive kidney disease among African Americans subjects and showed that ACEi appeared to be more effective than beta-blockers or dihydropyridine calcium channel blockers in GFR decline [52]. Our study supported the benefit of RAAS blockage on ESRD outcome in non-diabetic CKD patients and also revealed additional benefit of this drug group by reducing death before ESRD [53].

Our results found a very high risk of death after ESRD diagnosis which corresponded with the study from Chinese ESRD subjects that the risk of death was high as 50% after 5 years follow-up [54] and could not demonstrate the beneficial effect of RAAS on death after ESRD diagnosis. It was found that sudden cardiac death was the major cause of death among ESRD patients and the underlying mechanism may be cardiac arrthymia which contribute by bradycardia or asystole or ventricular tachycardia [55–57]. In addition, the post hoc analysis among the CHOICE (Choices for Healthy Outcomes In Caring for ESRD) cohort of 1041 incident dialysis patients reported that sudden cardiac death is common among patients with ESRD and that inflammation and malnutrition significantly increased its occurrence independent of traditional cardiovascular risk factors [58]. These different mechanisms may not associate with the protective effect from RAAS blockers.

Our study has some strengths. Firstly, we had a large number of subjects included in the analysis. To our knowledge, our study is the largest study assessing the efficacy of RAAS blockers on ESRD outcome compared to previous individual RCTs (enrolled 36–4912 subjects [9]) or previous individual observational studies (1094–20,152 subjects). In addition, most previous RCTs used the composite outcome of ESRD, doubling of serum creatinine, or death to increase the number of rare events as such ESRD. Secondly, data for medication use was from real clinical practice, where adherence is potentially lower than in RCTs; hence, this would tend to bias towards the null, making our results even more robust. Thirdly, although the best way to control for confounding is to use a randomized controlled trial, such evidence is not available in this case. A counterfactual analysis is thought to be one of the best ways to use observational data, in that it reduces the effect of confounders more than adjustment in a traditional regression model. This is due to a couple of reasons; 1) adjustment for varying combinations of confounders creates many cells, many of which may have sparse data. AIPW is a marginal method which is not affected by this problem and 2) adjustment for more and more confounders creates the potential for collider bias, in which adjustment for one confounder opens additional pathways for further confounding. Again, AIPW avoids this problem. Although this data is consistent with RCT evidence from other populations, evidence from RCTs within this population should be sought in order to confirm this finding. Results of our study should be useful for clinicians as well as health policy makers in the management of CKD patients while waiting for better evidence. As for our data, the overall RAAS blockade prescription rates were relatively low particularly in those patients with overt proteinuria. This reflected the lack of awareness of reno-protective effects of RAAS blockade among general practitioners and internists and should urgently raise a call for actions to increase such awareness.

Our study also had some limitations. This study was a retrospective cohort in which the data were retrieved from databases of routine practice. Data quality controls, standardized laboratory tests, and completeness of data were not as good as a purpose-designed prospective cohort. Data on adherence to RAAS blockade agents was not available. In addition, some other covariables including BMI, co-morbidity, treatments, and achievement of treatment targets were also not complete. Duration of RAAS use was captured crudely using 3 categories; there was insufficient data to model this as a time-varying covariate, which would have been more powerful.

## Conclusions

In conclusion, in subjects with diabetic or non-diabetic CKD, RAAS blockade therapy for 1 year or longer could prevent both CKD progression to ESRD and premature mortality in clinical practice setting. These findings suggest that in CKD patients, RAAS blockage may be the medication of choice for controlling blood pressure.

## Additional file

Additional file 1: Table S1. Estimation of average treatment model for ESRD in diabetic patients. **Table S2.** Estimation of average treatment model for ESRD in non-diabetic patients (RTF 343 kb)

## Abbreviations

AASK: African American Study of Kidney Disease and Hypertension
ACEi: Angiotensin converting enzyme inhibitors
ACR: Albumin-to-creatine ratio
AIPW: Augmented inverse-probability weighting
ARB: Angiotensin receptor blockers
ATE: Average treatment-effect
BMI: Body mass index
BUN: Blood urea nitrogen
CKD: Chronic kidney disease
CVD: Cardiovascular disease
DKD: Diabetic kidney disease
eGFR: Estimated glomerular filtration rate
ESRD: End-stage renal disease
FPG: Fasting plasma glucose
HDL: High density lipoprotein
KDIGO: Kidney Disease: Improving Global Outcome
NNT: Numbers needed-to-treat
POM: Potential-outcome mean
RAAS: Renin angiotensin aldosterone system
RD: Risk differences
RCT: Randomized controlled trials
UA: Urine analysis

## References

[1] Klag MJ, Whelton PK, Randall BL, Neaton JD, Brancati FL, Ford CE, Shulman NB, Stamler J (1996). Blood pressure and end-stage renal disease in men. N Engl J Med.

[2] Hsu CY, McCulloch CE, Darbinian J, Go AS, Iribarren C (2005). Elevated blood pressure and risk of end-stage renal disease in subjects without baseline kidney disease. Arch Intern Med.

[3] Brancati FL, Whelton PK, Randall BL, Neaton JD, Stamler J, Klag MJ (1997). Risk of end-stage renal disease in diabetes mellitus: a prospective cohort study of men screened for MRFIT. Multiple Risk Factor Intervention Trial. Jama.

[4] Pavkov ME, Bennett PH, Knowler WC, Krakoff J, Sievers ML, Nelson RG (2006). Effect of youth-onset type 2 diabetes mellitus on incidence of end-stage renal disease and mortality in young and middle-aged pima Indians. JAMA.

[5] Diamond JR (1991). Analogous pathobiologic mechanisms in glomerulosclerosis and atherosclerosis. Kidney Int Suppl.

[6] Siragy HM, Carey RM (2010). Role of the intrarenal renin-angiotensin-aldosterone system in chronic kidney disease. Am J Nephrol.

[7] Global, regional, and national age-sex specific all-cause and cause-specific mortality for 240 causes of death, 1990–2013: a systematic analysis for the Global Burden of Disease Study 2013. (1474-547X (Electronic)). 10.1016/S0140-6736(14)61682-2 PMC4340604 25530442

[8] KDOQI Clinical Practice Guideline for Diabetes and CKD: 2012 Update. (1523–6838 (Electronic)). 10.1053/j.ajkd.2012.07.005 23067652

[9] Vejakama P, Thakkinstian A, Lertrattananon D, Ingsathit A, Ngarmukos C, Attia J (2012). Reno-protective effects of renin-angiotensin system blockade in type 2 diabetic patients: a systematic review and network meta-analysis. Diabetologia.

[10] Balamuthusamy S, Srinivasan L, Verma M, Adigopula S, Jalandhara N, Hathiwala S, Smith E (2008). Renin angiotensin system blockade and cardiovascular outcomes in patients with chronic kidney disease and proteinuria: a meta-analysis. Am Heart J.

[11] Sharma P, Blackburn RC, Parke CL, McCullough K, Marks A, Black C (2011). Angiotensin-converting enzyme inhibitors and angiotensin receptor blockers for adults with early (stage 1 to 3) non-diabetic chronic kidney disease. Cochrane Database Syst Rev.

[12] Huang RS, Cheng YM, Zeng XX, Kim S, Fu P (2016). Renoprotective effect of the combination of Renin-angiotensin system inhibitor and Calcium Channel blocker in patients with hypertension and chronic kidney disease. Chin Med J.

[13] Suissa S, Hutchinson T, Brophy JM, Kezouh A (2006). ACE-inhibitor use and the long-term risk of renal failure in diabetes. Kidney Int.

[14] Hsu TW, Liu JS, Hung SC, Kuo KL, Chang YK, Chen YC, Hsu CC, Tarng DC (2014). Renoprotective effect of renin-angiotensin-aldosterone system blockade in patients with predialysis advanced chronic kidney disease, hypertension, and anemia. JAMA Intern Med.

[15] Feringa HH, Karagiannis SE, Chonchol M, Vidakovic R, Noordzij PG, Elhendy A, van Domburg RT, Welten G, Schouten O, Bax JJ (2007). Lower progression rate of end-stage renal disease in patients with peripheral arterial disease using statins or Angiotensin-converting enzyme inhibitors. J Am Soc Nephrol.

[16] Ahmed A, Fonarow GC, Zhang Y, Sanders PW, Allman RM, Arnett DK, Feller MA, Love TE, Aban IB, Levesque R (2012). Renin-angiotensin inhibition in systolic heart failure and chronic kidney disease. Am J Med.

[17] McAlister FA, Ezekowitz J, Tonelli M, Armstrong PW (2004). Renal insufficiency and heart failure: prognostic and therapeutic implications from a prospective cohort study. Circulation.

[18] Berger AK, Duval S, Manske C, Vazquez G, Barber C, Miller L, Luepker RV (2007). Angiotensin-converting enzyme inhibitors and angiotensin receptor blockers in patients with congestive heart failure and chronic kidney disease. Am Heart J.

[19] Tokmakova MP, Skali H, Kenchaiah S, Braunwald E, Rouleau JL, Packer M, Chertow GM, Moye LA, Pfeffer MA, Solomon SD (2004). Chronic kidney disease, cardiovascular risk, and response to angiotensin-converting enzyme inhibition after myocardial infarction: the survival and ventricular enlargement (SAVE) study. Circulation.

[20] Reinecke H, Matzkies F, Fobker M, Breithardt G, Schaefer RM (2005). Diabetic nephropathy, percutaneous coronary interventions, and blockade of the renin-angiotensin system. Cardiology.

[21] Gibney EM, Casebeer AW, Schooley LM, Cunningham F, Grover FL, Bell MR, McDonald GO, Shroyer AL, Parikh CR (2005). Cardiovascular medication use after coronary bypass surgery in patients with renal dysfunction: a national veterans administration study. Kidney Int.

[22] Ezekowitz J, McAlister FA, Humphries KH, Norris CM, Tonelli M, Ghali WA, Knudtson ML, Investigators A (2004). The association among renal insufficiency, pharmacotherapy, and outcomes in 6,427 patients with heart failure and coronary artery disease. J Am Coll Cardiol.

[23] So WY, Ozaki R, Chan NN, Tong PC, Ho CS, Lam CW, Ko GT, Chow CC, Chan WB, Ma RC *et al*: Effect of angiotensin-converting enzyme inhibition on survival in 3773 Chinese type 2 diabetic patients. Hypertension *(Dallas, Tex : 1979)* 2004, 44(3):294-299. 10.1161/01.HYP.0000137192.19577.c3 15249544

[24] Molnar MZ, Kalantar-Zadeh K, Lott EH, Lu JL, Malakauskas SM, Ma JZ, Quarles DL, Kovesdy CP (2014). Angiotensin-converting enzyme inhibitor, angiotensin receptor blocker use, and mortality in patients with chronic kidney disease. J Am Coll Cardiol.

[25] Luo Z, Gardiner Jc Fau - Bradley CJ, Bradley CJ: Applying propensity score methods in medical research: pitfalls and prospects. (1552–6801 (Electronic)). 10.1177/1077558710361486 PMC3268514 20442340

[26] McDonald S, Lambert J: The long arm of mentoring: a counterfactual analysis of natural youth mentoring and employment outcomes in early careers. (1573–2770 (Electronic)). 10.1007/s10464-014-9670-2 25145676

[27] Tararbit K, Lelong N Fau - Houyel L, Houyel L Fau - Bonnet D, Bonnet D Fau - Goffinet F, Goffinet F Fau - Khoshnood B, Khoshnood B: Assessing the role of multiple pregnancies in the association between tetralogy of Fallot and assisted reproductive techniques: a path-analysis approach. (1750–1172 (Electronic)). 10.1186/1750-1172-9-27 PMC3933480 24555734

[28] Schofield DJ, Callander EJ, Shrestha RN, Passey ME, Kelly SJ, Percival R: Multiple chronic health conditions and their link with wealth assets. (1464-360X (Electronic)). 10.1093/eurpub/cku134 25192707

[29] Vejakama P, Ingsathit A Fau - Attia J, Attia J Fau - Thakkinstian A, Thakkinstian A: Epidemiological study of chronic kidney disease progression: a large-scale population-based cohort study. (1536–5964 (Electronic)). 10.1097/MD.0000000000000475 PMC4602951 25634196

[30] Levey AS, Stevens LA, Schmid CH, Zhang YL, Castro AF, Feldman HI, Kusek JW, Eggers P, Van Lente F, Greene T (2009). A new equation to estimate glomerular filtration rate. Ann Intern Med.

[31] Levin A, Stevens PE: Summary of KDIGO 2012 CKD Guideline: behind the scenes, need for guidance, and a framework for moving forward. (1523–1755 (Electronic)). 10.1038/ki.2013.444 24284513

[32] Rubin DB, Schenker N: Multiple imputation in health-care databases: an overview and some applications. (0277–6715 (Print)). 10.1002/sim.4780100410 2057657

[33] White IR, Royston P Fau - Wood AM, Wood AM: Multiple imputation using chained equations: Issues and guidance for practice. (1097–0258 (Electronic)). 10.1002/sim.4067 21225900

[34] van Buuren S, Boshuizen Hc Fau - Knook DL, Knook DL: Multiple imputation of missing blood pressure covariates in survival analysis. (0277–6715 (Print)). 10.1002/(sici)1097-0258(19990330)18:6<681::aid-sim71>3.0.co;2-r 10204197

[35] Cerulli G (2014). Ivtreatreg: a command for fitting binary treatment models with heterogeneous response to treatment and unobservable selection. Stata J.

[36] Cerulli G (2014). Treatrew: a user-written command for estimating average treatment effects by reweighting on the propensity score. Stata J.

[37] StataCorp.: Stata statistical software: release 14. In: Treatment-effects reference manual. College Station**,** TX**:** StataCorp LP. 2014.

[38] Standards of medical care in diabetes--2014. (1935–5548 (Electronic)).

[39] Yacoub R, Campbell KN: Inhibition of RAS in diabetic nephropathy. (1178–7058 (Electronic)). 10.2147/IJNRD.S37893 PMC4403683 25926752

[40] Lewis G Fau - Maxwell AP, Maxwell AP: Risk factor control is key in diabetic nephropathy. (0032–6518 (Print)). 24689163

[41] Ferrari R, Boersma E: The impact of ACE inhibition on all-cause and cardiovascular mortality in contemporary hypertension trials: a review. (1744–8344 (Electronic)). 10.1586/erc.13.42 23750680

[42] Cockcroft JR: ACE inhibition in hypertension: focus on perindopril. (1175–3277 (Print)). 10.2165/00129784-200707050-00001 17953470

[43] Vejakama P, Ingsathit A, Attia J, Thakkinstian A (2015). Epidemiological study of chronic kidney disease progression: a large-scale population-based cohort study. Medicine (Baltimore).

[44] Muskiet MH, Smits MM, Morsink LM, Diamant M (2014). The gut-renal axis: do incretin-based agents confer renoprotection in diabetes?. Nat Rev Nephrol.

[45] Cooper ME (2001). Interaction of metabolic and haemodynamic factors in mediating experimental diabetic nephropathy. Diabetologia.

[46] Fioretto P, Mauer M (2007). Histopathology of diabetic nephropathy. Semin Nephrol.

[47] Parving HH, Lehnert H, Brochner-Mortensen J, Gomis R, Andersen S, Arner P (2001). The effect of irbesartan on the development of diabetic nephropathy in patients with type 2 diabetes. N Engl J Med.

[48] Lewis EJ, Hunsicker LG, Clarke WR, Berl T, Pohl MA, Lewis JB, Ritz E, Atkins RC, Rohde R, Raz I (2001). Renoprotective effect of the angiotensin-receptor antagonist irbesartan in patients with nephropathy due to type 2 diabetes. N Engl J Med.

[49] Brenner BM, Cooper ME, de Zeeuw D, Keane WF, Mitch WE, Parving HH, Remuzzi G, Snapinn SM, Zhang Z, Shahinfar S (2001). Effects of losartan on renal and cardiovascular outcomes in patients with type 2 diabetes and nephropathy. N Engl J Med.

[50] Remuzzi G, Perico N, Macia M, Ruggenenti P (2005). The role of renin-angiotensin-aldosterone system in the progression of chronic kidney disease. Kidney Int Suppl.

[51] Jafar TH, Schmid CH, Landa M, Giatras I, Toto R, Remuzzi G, Maschio G, Brenner BM, Kamper A, Zucchelli P (2001). Angiotensin-converting enzyme inhibitors and progression of nondiabetic renal disease. A meta-analysis of patient-level data. Ann Intern Med.

[52] Wright JT, Bakris G, Greene T, Agodoa LY, Appel LJ, Charleston J, Cheek D, Douglas-Baltimore JG, Gassman J, Glassock R (2002). Effect of blood pressure lowering and antihypertensive drug class on progression of hypertensive kidney disease: results from the AASK trial. JAMA.

[53] Qin Y, Chen T, Chen Q, Lv JY, Qi N, Wu C, He J: The effect of angiotensin-converting enzyme inhibitor/angiotensin receptor blocker use on mortality in patients with chronic kidney disease: a meta-analysis of observational studies. Pharmacoepidemiol Drug Saf. 2016;25(5):503-11. 10.1002/pds.3941 26786323

[54] Wang AY, Lam CW, Chan IH, Wang M, Lui SF, Sanderson JE: Sudden cardiac death in end-stage renal disease patients: a 5-year prospective analysis. Hypertension *(Dallas, Tex : 1979)* 2010, 56(2):210-216. 10.1161/HYPERTENSIONAHA.110.151167 20606110

[55] Mavrakanas TA, Charytan DM (2016). Cardiovascular complications in chronic dialysis patients. Curr Opin Nephrol Hypertens.

[56] Wan C, Herzog CA, Zareba W, Szymkiewicz SJ (2014). Sudden cardiac arrest in hemodialysis patients with wearable cardioverter defibrillator. Ann Noninvasive Electrocardiol.

[57] Wong MC, Kalman JM, Pedagogos E, Toussaint N, Vohra JK, Sparks PB, Sanders P, Kistler PM, Halloran K, Lee G (2015). Bradycardia and asystole is the predominant mechanism of sudden cardiac death in patients with chronic kidney disease. J Am Coll Cardiol.

[58] Parekh RS, Plantinga LC, Kao WH, Meoni LA, Jaar BG, Fink NE, Powe NR, Coresh J, Klag MJ (2008). The association of sudden cardiac death with inflammation and other traditional risk factors. Kidney Int.

