# Supplementary material for: Treatment effects of renin-angiotensin aldosterone system blockade on kidney failure and mortality in chronic kidney disease patients
Source: BMC Nephrol. 2017 Nov 29;18:342. doi: 10.1186/s12882-017-0753-9 (PMC5706339; doi:10.1186/s12882-017-0753-9)
Supplement: Additional file 1: Table S1. — Estimation of average treatment model for ESRD in diabetic patients. Table S2. Estimation of average treatment model for ESRD in non-diabetic patients (RTF 343 kb) [file 12882_2017_753_MOESM1_ESM.rtf]

Additional file 1: Table S1 Estimation of average treatment model for ESRD in diabetic patients 
Additional file 1: Table S2 Estimation of average treatment model for ESRD in non-diabetic patients 


Additional file 1: Table S1. Estimation of average treatment model for ESRD in diabetic patients  
Model	Factors	coef	SE	Z	P>|Z|	[95% Conf.	Interval]	
ATE	RAAS1 vs non-RAAS	-0.0100	0.0176	-0.566	0.572	-0.0445	0.0246	
	RAAS2 vs non-RAAS	-0.0712	0.0084	-8.459	0.000	-0.0877	-0.0547	
POmean	Non-RAAS	0.1999	0.0042	47.543	0.000	0.1917	0.2082	
OME0	Age	0.0075	0.0019	3.950	0.000	0.0038	0.0112	
	Sex	0.0403	0.0463	0.870	0.384	-0.0504	0.1310	
	BMI	-0.0461	0.0054	-8.500	0.000	-0.0568	-0.0355	
	Hypertension	0.2162	0.0428	5.053	0.000	0.1323	0.3001	
	CVD	0.0166	0.0665	0.250	0.803	-0.1137	0.1469	
	HDL	-0.0068	0.0019	-3.565	0.000	-0.0106	-0.0031	
	Albumin							
	A1	0.4347	0.0569	7.641	0.000	0.3232	0.5462	
	A2	1.1135	0.0468	23.787	0.000	1.0218	1.2053	
OME1	Age	-0.0037	0.0067	-0.547	0.584	-0.0168	0.0095	
	Sex	0.4019	0.1393	2.885	0.004	0.1289	0.6749	
	BMI	-0.0430	0.0197	-2.187	0.029	-0.0816	-0.0045	
	Hypertension	0.3877	0.1879	2.064	0.039	0.0195	0.7560	
	CVD	-0.0081	0.1940	-0.042	0.967	-0.3883	0.3722	
	HDL	-0.0032	0.0063	-0.513	0.608	-0.0155	0.0090	
	Albumin	0.0000	0.0000			0.0000	0.0000	
	   A1	0.4136	0.2105	1.965	0.049	0.0011	0.8261	
	   A2	0.8144	0.1563	5.210	0.000	0.5080	1.1208	
OME2	Age	0.0030	0.0050	0.605	0.545	-0.0068	0.0128	
	Sex	-0.1280	0.1046	-1.224	0.221	-0.3330	0.0770	
	BMI	-0.0326	0.0130	-2.509	0.012	-0.0581	-0.0071	
	Hypertension	0.3746	0.1205	3.108	0.002	0.1384	0.6108	
	CVD	0.0405	0.1398	0.290	0.772	-0.2335	0.3146	
	HDL	-0.0129	0.0043	-2.958	0.003	-0.0214	-0.0043	
	Albumin	0.0000	0.0000			0.0000	0.0000	
	   A1	0.9553	0.1323	7.223	0.000	0.6961	1.2145	
	   A2	1.4563	0.1140	12.779	0.000	1.2329	1.6796	
TME1	Age	-0.0038	0.0049	-0.782	0.434	-0.0134	0.0058	
	Sex	0.3449	0.1059	3.258	0.001	0.1374	0.5525	
	BMI	-0.0008	0.0121	-0.069	0.945	-0.0245	0.0228	
	Hypertension	1.7414	0.1108	15.714	0.000	1.5242	1.9587	
	CVD	0.0116	0.1423	0.081	0.935	-0.2673	0.2904	
	HDL	-0.0017	0.0043	-0.389	0.697	-0.0102	0.0068	
	Albumin	0.0000	0.0000			0.0000	0.0000	
	   A1	-0.2661	0.1346	-1.977	0.048	-0.5299	-0.0023	
	   A2	0.0887	0.1195	0.742	0.458	-0.1456	0.3229	
	GFR	-0.0127	0.0033	-3.855	0.000	-0.0191	-0.0062	
TME2	Age	-0.0074	0.0023	-3.240	0.001	-0.0119	-0.0029	
	Sex	0.1813	0.0505	3.589	0.000	0.0823	0.2803	
	BMI	0.0389	0.0056	6.904	0.000	0.0278	0.0499	
	Hypertension	1.9027	0.0499	38.134	0.000	1.8049	2.0005	
	CVD	-0.1027	0.0722	-1.423	0.155	-0.2441	0.0387	
	HDL	0.0075	0.0019	3.866	0.000	0.0037	0.0113	
	Albumin	0.0000	0.0000			0.0000	0.0000	
	   A1	-0.1507	0.0593	-2.541	0.011	-0.2670	-0.0345	
	   A2	0.0138	0.0586	0.235	0.814	-0.1010	0.1286	
	GFR	0.0158	0.0012	13.158	0.000	0.0135	0.0182	
ATE, average treatment effect; LL, lower limit; OME0-2, potential outcome equations for non-RAAS, RAAS1 and RAAS2; POM, potential outcome mean; RAAS1, duration use of 0.25-1 year; RAAS2, duration use of > 1year; RD, risk difference; RR, rate ratio; TME1-2, treatment effect equation for RAAS1 and RAAS2; UL, upper limit


Additional file 1: Table S2.  Estimation of average treatment model for ESRD in non-diabetic patients 
Model 	Factors	coef	SE	Z	P>|Z|	[95% Conf.	Interval]	
ATE	RAAS1 vs non-RAAS	-0.0205	0.0336	-0.611	0.541	-0.0863	0.0453	
	RAAS2 vs non-RAAS	-0.0674	0.0277	-2.436	0.015	-0.1216	-0.0132	
POmean	Non-RAAS	0.1812	0.0034	52.789	0.000	0.1745	0.1880	
OME0	Age	0.0100	0.0014	7.026	0.000	0.0072	0.0128	
	Sex	-0.2419	0.0382	-6.328	0.000	-0.3169	-0.1670	
	BMI	-0.0682	0.0049	-13.789	0.000	-0.0778	-0.0585	
	Hypertension	-0.2096	0.0460	-4.557	0.000	-0.2998	-0.1194	
	CVD	-0.1451	0.0599	-2.423	0.015	-0.2624	-0.0277	
	HDL	-0.0239	0.0024	-10.102	0.000	-0.0285	-0.0193	
	Albumin	0.0000	0.0000			0.0000	0.0000	
	A1	0.5320	0.0459	11.587	0.000	0.4420	0.6220	
	A2	0.9337	0.0444	21.051	0.000	0.8468	1.0207	
OME1	Age	-0.0185	0.0089	-2.078	0.038	-0.0359	-0.0010	
	Sex	0.1567	0.1971	0.795	0.426	-0.2295	0.5430	
	BMI	-0.1157	0.0256	-4.518	0.000	-0.1659	-0.0655	
	Hypertension	0.1085	0.2952	0.367	0.713	-0.4702	0.6871	
	CVD	-0.2331	0.2812	-0.829	0.407	-0.7842	0.3180	
	HDL	-0.0234	0.0095	-2.461	0.014	-0.0421	-0.0048	
	Albumin	0.0000	0.0000			0.0000	0.0000	
	A1	0.5690	0.2782	2.045	0.041	0.0237	1.1142	
	A2	1.0160	0.2289	4.438	0.000	0.5673	1.4646	
OME2	Age	-0.0057	0.0095	-0.594	0.553	-0.0244	0.0130	
	Sex	0.0958	0.2127	0.451	0.652	-0.3211	0.5127	
	BMI	-0.0774	0.0287	-2.699	0.007	-0.1336	-0.0212	
	Hypertension	-0.3886	0.3380	-1.150	0.250	-1.0509	0.2738	
	CVD	0.1288	0.2584	0.499	0.618	-0.3777	0.6354	
	HDL	-0.0305	0.0104	-2.944	0.003	-0.0509	-0.0102	
	Albumin	0.0000	0.0000			0.0000	0.0000	
	A1	0.6896	0.2945	2.342	0.019	0.1124	1.2668	
	A2	1.4978	0.2332	6.424	0.000	1.0408	1.9548	
TME1	Age	0.0006	0.0045	0.142	0.887	-0.0081	0.0094	
	Sex	0.1837	0.0984	1.866	0.062	-0.0092	0.3765	
	BMI	0.0149	0.0118	1.261	0.207	-0.0082	0.0380	
	Hypertension	2.6978	0.1492	18.077	0.000	2.4053	2.9903	
	CVD	0.2573	0.1348	1.909	0.056	-0.0069	0.5215	
	HDL	0.0112	0.0045	2.511	0.012	0.0025	0.0200	
	Albumin	0.0000	0.0000			0.0000	0.0000	
	A1	-0.1796	0.1411	-1.272	0.203	-0.4562	0.0971	
	A2	-0.0279	0.1488	-0.187	0.851	-0.3195	0.2637	
	GFR	-0.0110	0.0029	-3.767	0.000	-0.0167	-0.0053	
TME2	Age	-0.0070	0.0027	-2.565	0.010	-0.0124	-0.0017	
	Sex	0.1629	0.0600	2.714	0.007	0.0452	0.2805	
	BMI	0.0288	0.0072	3.997	0.000	0.0147	0.0430	
	Hypertension	3.5203	0.1167	30.168	0.000	3.2916	3.7490	
	CVD	0.4717	0.0824	5.727	0.000	0.3103	0.6332	
	HDL	0.0113	0.0027	4.117	0.000	0.0059	0.0166	
	Albumin	0.0000	0.0000			0.0000	0.0000	
	A1	-0.4354	0.0903	-4.821	0.000	-0.6125	-0.2584	
	A2	-0.3646	0.1005	-3.629	0.000	-0.5616	-0.1677	
	GFR	0.0096	0.0016	6.160	0.000	0.0065	0.0126	
ATE, average treatment effect; LL, lower limit; OME0-2, potential outcome equations for non-RAAS, RAAS1 and RAAS2; POM, potential outcome mean; RAAS1, duration use of 0.25-1 year; RAAS2, duration use of > 1year; RD, risk difference; RR, rate ratio; TME1-2, treatment effect equation for RAAS1 and RAAS2; UL, upper limit
